# A comprehensive modelling approach to estimate the transmissibility of coronavirus and its variants from infected subjects in indoor environments

**DOI:** 10.1038/s41598-022-17693-z

**Published:** 2022-08-19

**Authors:** S. Anand, Jayant Krishan, B. Sreekanth, Y. S. Mayya

**Affiliations:** 1 Health Physics Division, Bhabha Atomic Research Centre, Mumbai, 400 085 India; 2 Radiation Safety and Systems Division, Bhabha Atomic Research Centre, Mumbai, 400 085 India; 3 Homi Bhabha National Institute, Mumbai, 400 094 India; 4 Department of Chemical Engineering, Indian Institute of Technology Bombay, Mumbai, 400 076 India

**Keywords:** Viral infection, Risk factors, Applied mathematics

## Abstract

A central issue in assessing the airborne risk of COVID-19 infections in indoor spaces pertains to linking the viral load in infected subjects to the lung deposition probability in exposed individuals through comprehensive aerosol dynamics modelling. In this paper, we achieve this by combining aerosol processes (evaporation, dispersion, settling, lung deposition) with a novel double Poisson model to estimate the probability that at least one carrier particle containing at least one virion will be deposited in the lungs and infect a susceptible individual. Multiple emission scenarios are considered. Unlike the hitherto used single Poisson models, the double Poisson model accounts for fluctuations in the number of carrier particles deposited in the lung in addition to the fluctuations in the virion number per carrier particle. The model demonstrates that the risk of infection for 10-min indoor exposure increases from 1 to 50% as the viral load in the droplets ejected from the infected subject increases from 2 × 10^8^ to 2 × 10^10^ RNA copies/mL. Being based on well-established aerosol science and statistical principles, the present approach puts airborne risk assessment methodology on a sound formalistic footing, thereby reducing avoidable epistemic uncertainties in estimating relative transmissibilities of different coronavirus variants quantified by different viral loads.

## Introduction

More than 8.2 billion vaccine doses have been administered across 182 countries worldwide to prevent the COVID-19 disease, and ~ 48% of the global population is fully vaccinated against SARS-CoV-2 virus (https://covid19.who.int). The overall pattern of the coronavirus pandemic so far has been a series of COVID-19 waves in several parts of the globe. The outbreak peaks were each caused by early strains of SARS-CoV-2, the Beta variant, or the Delta variant. The recent increase in the number of infections at a faster rate is due to the new variant, Omicron^1–3^. Thus, the emergence of pandemic has seen an increasing transmissibility associated with each variant/phase of the disease. Since new mutant variants of SARS-CoV-2 virus have different infectious characteristics, it is very important to study the factors that control indoor infection risk using comprehensive model^4–6^. This will help in understanding the disease spread and infection risk related to low and medium risk events apart from superspreading scenarios.

Despite initial epistemic uncertainty, the aerosol route is now recognized as the principal path of transmission of COVID-19. Several infection risk models are developed to quantify the risk posed by airborne infectious diseases through aerosol route in the indoor environments^7–13^. These single hit models are based on the deposition of a single virion/unit/colony as the initiating factor of the disease, which relegates the viral content of the droplets to the secondary factors such as droplet size, and virion concentration in saliva or mucus. In these models, inhaled droplet volume is treated as a continuous variable and Poisson fluctuations are assigned to the events of virus incorporation in the total inhaled droplet volume. However, this lacks generality; just as the virions are discrete, droplet inhalation is also a discrete process. In order to get one virion deposited in the lung, one must inhale at least one droplet. This calls for a double Poisson probability approach which has been incorporated successfully in the present model.

Another important aspect of the risk model is coupling of the droplet evaporation process with other dynamical processes such as dispersion in the indoor space and gravitational settling. The precise modelling of evaporation of droplets containing non-volatile salts is a complex process, and hence many risk models assume a constant final diameter of the droplets (ex. ~ 50% of their initial value at the emission) and the residue distribution is simply a numerical contraction of the original droplet distribution via a scaling law based on residue concentration. Instead, the present model takes into account the fact that the larger droplets might not have dried fully and also the fact that smaller droplets might be in Kohler equilibrium. The present seamless coupled modelling approach eliminates the dichotomous approach of assigning the settling velocities either only to dry/wet particles. In this model, the dispersion mechanism is treated by the ventilation dependent turbulent diffusion process in which the diffusion coefficient is phenomenologically related to the air-exchange rate. The deposition of the airborne virus-laden droplets in the human respiratory tract is another important process and demands attention since it is a strong function of droplet size and dimension of the parts of the respiratory system. Also, there is growing evidence that different variants infect different parts of the respiratory system; for example, recent studies show that the Omicron variant infects and multiplies faster than the original and Delta variants in human bronchus^14^. In the present work, both bronchial and pulmonary deposition events are taken into consideration through empirical relation based on the standard ICRP lung deposition model^15^.

A comparison of risk estimation models from the literature^7–13,16,17^ along with the present model is given in the Supplementary Table S1. Most of them are based on quanta generation rate and Wells–Riley “one-hit” risk hypothesis (single Poisson model for infection risk), and concerned more about superspreading events. Although qualitatively, there is an understanding about the impact of viral load on the infection risk, the following question remains unanswered—how enhanced viral load (infectivity of the disease) increases the transmissibility of the disease, mediated by aerosol route? The missing link can only be addressed by coupling the viral load to the aerosol mechanics.

In this study, we formulate a comprehensive model by combining the detailed aerosol dynamics with a novel double Poisson model that accounts for fluctuations in the number of carrier particles deposited in the lung in addition to the fluctuations in the virion number per carrier particle. Using the above formulation, the infection risk is evaluated as a function of viral load for a weighted scenario of expiratory events, and consideration of protection barriers both by the infected and the target subject. The present approach, being based on well-established aerosol principles and statistical methods, places airborne risk assessment on a solid formalistic foundation by eliminating approximation methodologies. Thus, the estimated transmissibility observed in the case of Delta and Omicron variants, which produce a higher viral load than older wild SARS-CoV-2 variants, will have lower epistemic uncertainty. The results are discussed in the following sections.

## Results and discussion

### Model

The present comprehensive model combines the detailed aerosol dynamics with a novel double Poisson model to estimate the probability that at least one carrier particle containing at least one virion will be deposited in the lungs. This model recognizes not only the discreteness of virions and their fluctuations but also that of the inhaled residues/droplets which vector them and hence, introduces fluctuations in the entire size spectrum^18–20^. The aerosol dynamics accounts for evaporation, residue formation, room dispersion, settling, plate-out and deposition in the respiratory tract of the inhaling subject.

**Figure 1:**
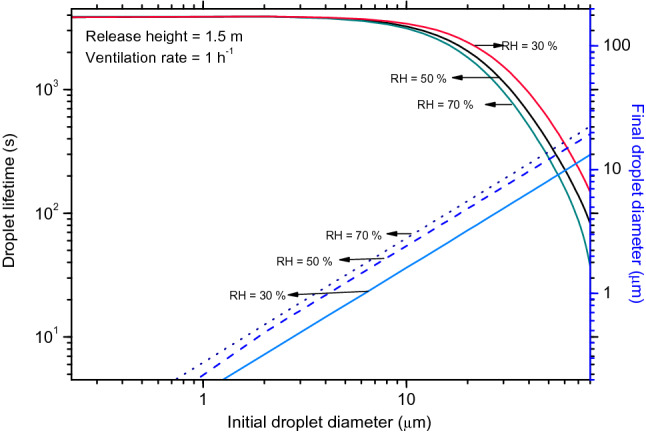
Lifetime of droplets in a typical indoor environment.

In the present work, the falling-to-mixing-plate-out model^21^ is implemented, which allows a droplet's residence time (τ) to smoothly transition from a gravity-dominated (larger particles, diameter > 50 µm, τ < 100 s) to a turbulence-dominated (small particle, diameter < 5 µm, τ > 3000 s) regime as shown in Fig. 1. It is worth mentioning that turbulent mixing extends the particle residence time for droplets of intermediate size. The variation of droplet lifetime with RH is significant only for large particles of diameter in the range of 20–80 µm, mainly due to evaporation and gravitational settling in this size regime. The study results show that the lifetime of virusols in the indoor environment is determined mainly by deposition; however, viral deposition in the lungs is entirely determined by viral load and aerosol physics. The reciprocal of the residence time of virus laden droplets to reach a given risk is an important parameter used to estimate the rate of propagation/transmissibility.

### Single-hit risk and reproduction number

The present study attempts to calculate the exposure time required to achieve a tangible single-hit risk for a given expiratory event as well as the event reproduction number (${R}_{e}$) for the given input parameters. Coughing^18,22,23^ and sneezing^18^ will be specific to the sick and symptomatic patients, although breathing^23^ and speaking^18,20,23^ are normal expiratory processes relevant to all subjects. Table 1 lists the parameters of expiratory emission^18,23,24^, such as droplet size distribution, frequency of emission, virion concentration in emitted droplets, etc.

**Table 1:** Emission characteristics of expiratory events.

Expiratory events	Size distribution parameters	Number release rate^a^	References
Breathing	CMD = 1.6 μm; GSD = 1.3	14 s^−1^ (continuous)	Johnson et al.^23^
Coughing	CMD = 14 μm; GSD = 2.6	28 s^−1^ (10 cough/h)	Duguid^18^
Speaking	CMD = 4 μm; GSD = 1.6	270 s^−1^ (5 min/h)	Johnson et al.^23^ Alsved et al.^24^
Sneezing	GM—8.1 μm; GSD = 2.3	2778 s^−1^ (10 sneezes/h)	Duguid^18^

^a^Long-time averaged droplet release rate.

**Figure 2:**
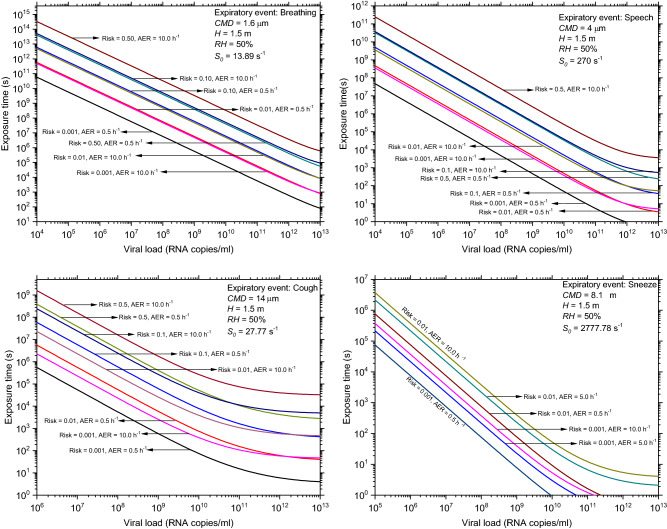
Exposure time as a function of viral load for a given infection risk and ventilation rate in the indoor environment.

For each expiratory event, numerical computations are used to determine the exposure time for different risk levels (0.1%, 1%, 10%, and 50%) and AERs (0.5–10 h^−1^). In the exposure time calculations, it is assumed that the emissions are continuous with the given rate and the value is estimated for a given risk. The model findings (Fig. 2) reveal that, up to a critical viral load, the exposure duration decreases linearly with the viral load in the log–log graph. Although the findings are not shown here, the slope of the linear component increases with emission rate (*S*_0_). The critical viral load in this case is, 10^13^ #/mL for breathing, 10^11^ #/mL for coughing, 10^10^ #/mL for sneezing, 10^12^ #/mL for speaking for a risk of 0.1%. Beyond this critical viral load, the risk becomes a constant or invariant with respect to the viral load. These results also show that the risk is strongly dependent on the emission rate; for example, if the particle emission rate of 1000 #/s for speech^20^ is considered, then the risk will increase in that proportion against the risk value estimated for 270 #/s^23,24^.

**Figure 3:**
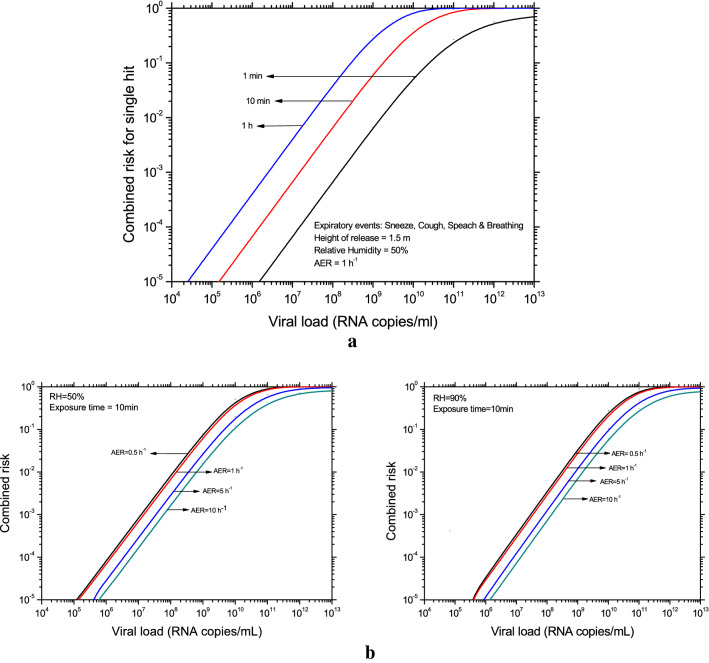
(**a**) Variation of single hit risk for susceptible persons as a function of viral load for different times of exposure. (**b**) Variation of single hit risk for susceptible persons as a function of viral load for RH and AER.

Alternative to the exposure time estimates, the single-hit risk (double Poisson model) is estimated under the influence of all the four expiratory events occurring simultaneously at given emission rates. The joint risk probability is then given by,

1 $${R}^{^{\prime}}=1-{P}_{0,B}\times {P}_{0,Sp}\times {P}_{0,C}\times {P}_{0,Sn,}$$

where ${P}_{0,B}=exp\left({-N}_{d}\left[1-exp\left(-{n}_{v}\right)\right]\right)$ is the probability of zero-hit for the breathing expiratory process, ${N}_{d}$ is the typical number of droplets inhaled by a person, ${n}_{v}$ is the average number of virions contained in a droplet, the suffices *Sp*, *C* and *Sn* denotes speaking, coughing and sneezing events respectively. It is to be noted that the transmissibility of a virus is measured via single-hit risk probability, dominated by the aerosol route of exposure. Also, it has been argued often that the transmissibility of the virus is linked with the viral load^3,25,26^, and hence, the risk of transmission to a susceptible individual is estimated as a function of viral load for specified exposure times (Fig. 3).

Numerical results (Fig. 3a) show that the risk is less than 1% for viral loads < 10^8^ RNA copies/mL for 1-h exposure period. But the risk rapidly approaches a higher value (ex. 50% for 10^10^ RNA copies/mL and 10-min exposure), which demonstrates the high transmissibility of Delta and possibly Omicron variants which are reported to give rise to higher viral loads^27–31^ (Table 2). Thus, the present study clearly demonstrates the risk dependence on the viral load irrespective of variants. The model also explores the effect of ventilation rate on indoor infection risks (Fig. 3b). When the air-exchange rate is increased from 0.5 to 10 h^−1^ for a 10-min exposure time, the single-hit risk decreases approximately by an order. This is primarily due to the elimination of airborne viruses from the indoor environment via ventilation. However, when viral load increases, the effect of enhancing ventilation reduces because smaller particles contribute to the risk as well. The ambient RH has only a minor impact on the risk; higher RH leads to larger final droplet sizes, which reduces their lifetime and therefore infection risk, as seen in Fig. 3b.

**Table 2:** Typical viral load of SARS-CoV-2 variants^27–31^.

SARS-CoV-2 variant	Viral load (RNA copies/mL)
Wild	~ 10^5^–10^8^
Delta	~ 10^6^–10^9^
Omicron	~ 10^6^–10^9.5^

**Figure 4:**
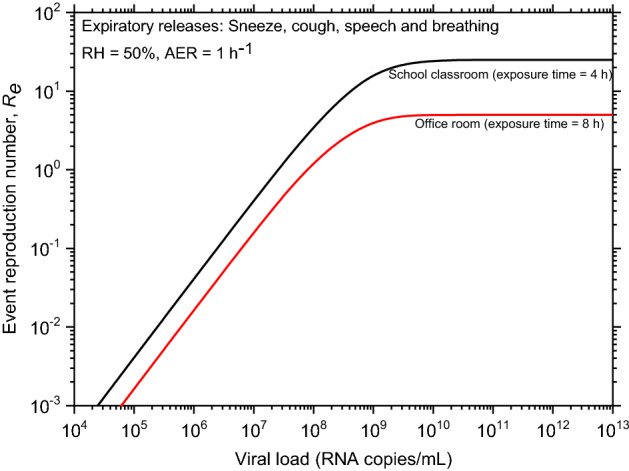
Event reproduction number as a function of viral load for two different indoor environments and exposure conditions.

Another essential metric to describe infection risk is the event reproduction number (*R*_e_), which is computed by multiplying the infection risk during the exposure time of each susceptible person by the number of susceptible people exposed for a specific exposure scenario. The following three scenarios are studied in this work to demonstrate how the model can be used: (a) 25 students in a classroom with an infected subject exposed for 4 h; (b) 4 employees in an office environment with an infected subject exposed for 8 h; (c) outbreak in a restaurant in Guangzhou, China. In the first scenario, the *R*_e_ value approaches 2 when the viral load of the infected person in the classroom exceeds 5 × 10^7^ #/mL, as shown in the results; also, the *R*_e_ value shall remain $\le$ 1 if the viral load is less than 2.5 × 10^7^ #/mL for the given input and environmental parameters, as shown in Fig. 4. Similarly, if the viral load is $\le$ 7 × 10^7^ #/mL for the given exposure conditions in an office setting, the *R*_e_ value will be $\le$ 1 in the second exposure scenario.

**Figure 5:**
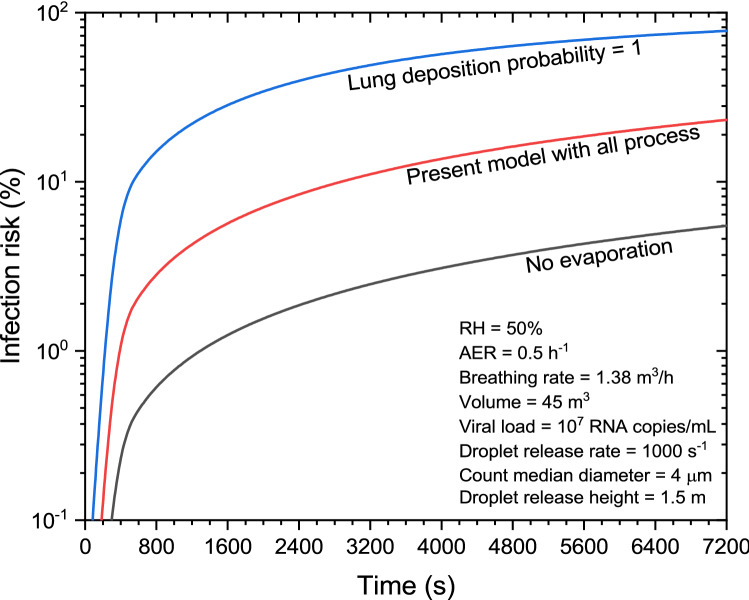
Infection risk as a function of exposure time for the outbreak at a restaurant.

The third case is a recognised outbreak^16,32^ in which a patient from an epidemic site had lunch in a restaurant of volume ~ 435 m^3^, with a floor area of 145 m^2^. To compare the present study results with the literature values, the input parameters for this superspreading event from Buonanno et al.^16^ are considered. At the time of the presence of the case patient with a viral load of 10^7^ RNA copies/mL, around 83 diners along with 8 staff members were present at the restaurant, and later some members of three families, who had lunch at the adjacent tables were found to be infected^16,32^. Considering speech as the continuous expiratory process, the infection risk is estimated as a function of exposure time (Fig. 5). The infection risk due to the presence of the case patient in a limited volume of ~ 45 m^3^ (volume encompassing the table of case patient and other adjacent tables) is estimated as 23% for 2-h exposure period, i.e., ~ (2–3) persons would be infected through aerosol transmission route (11 persons were present in this limited volume). This risk estimate is roughly half of the stated value in the literature^16^, which can be attributed to the differences in modelling because the input parameters are the same. If the complete volume of 435 m^3^ with 91 susceptible persons is considered instead of 45 m^3^, then the infection risk is reduced to ~ 2% and ~ 2 people would be infected. The decrease in risk value owing to an increase in indoor volume clearly demonstrates the dependence of the input parameter selection. The findings show that the model can be used to evaluate infection risk in low and medium risk events, including superspreading events. Thus, the new double Poissonian formalism combined with the comprehensive aerosol dynamics model introduced here makes a significant value addition to the subject of risk evaluation of airborne diseases.

From Fig. 5, it is observed that if droplet evaporation is neglected, the residual size will be higher, leading to larger removal by gravity. Hence, this would lead to lesser airborne droplet concentration and infection risk; i.e., 5.5% for this wet droplet distribution case compared to ~ 23% (if dynamic evaporation is considered in the model) at the end of 2-h exposure scenario. Another effect related to particle deposition in the respiratory system, if lung deposition model as given by ICRP^15^ is replaced by a lung deposition probability of 1 (i.e., 100% deposition after inhalation), the estimated infection risk increases by ~ 3.4 times, since a larger number of droplets are deposited in the respiratory tract leading to a higher value of infection risk. The risk value in this case is, ~ 78% when compared to the standard model study value of ~ 23%.

These findings imply that if the viral load is less than a certain value or if the contact period is limited for the specified emission and indoor settings, the event reproduction number will remain less than one. Alternatively, the limit on the number of people can also be estimated using the present approach for a given virus variant and the exposure duration. Hence, these studies can be used as a tool to aid decision/policy making as the spread of the disease can be directly predicted based on the viral load and other physically measurable input parameters.

## Conclusion

In this study, we apply a comprehensive approach by combining aerosol dynamics with a novel double Poisson risk model to obtain the crucial relationship between viral load and the transmissibility of respiratory viruses in the indoor environment. This is accomplished by considering the aerosol dynamics of evaporating droplets as well as fluctuations in the virion deposition probability in the lungs (both virions and droplets). The model accounts for several factors influencing the infection risk, such as the infected subject's mode of emission, droplet characteristics, indoor environment parameters (air humidity, ventilation, and turbulence), and protection factors (mask, air cleaners). At a formulation level, the present work introduces a paradigm shift through a new double Poissonian approach as opposed to the earlier single Poisson model for estimating the inhalation risk via the aerosol route across a wide range of exposure conditions.

Model results show that as the viral load increases, a saturation effect occurs, i.e. a larger fraction of droplets are virus laden and hence, the entire expiratory ejecta has the potential to contribute to the transmission of the disease. For a four-hour exposure in a classroom with 25 students, the event reproduction number is estimated to be less than 1 for viral loads ≤ 3 × 10^7^ RNA copies/mL and is more than 2 for viral loads > 10^8^ RNA copies/mL. This critical dependence of risk on the viral load, explains the crucial observations of the relatively higher transmission rate of Delta and Omicron variants via the viral load factor. Among various parameters examined, this model shows a dominating effect of the viral load, which is related to the variant type, on the risk of transmitting the disease to a susceptible member in a cohort.

The model can seamlessly handle cases of ultra-low risk (< 1%), as well as those approaching unity (superspreading events), because it includes fluctuations in both the virion content in a droplet and deposition probability in the lung through a novel double Poisson model. Finally, the study puts into perspective the effectiveness of technologies such as masks, air cleaners, and external ventilation in regulating infection risk. This, especially, is a desirable feature of the model as it will be helpful in assessing the cost-effectiveness of the technology deployment.

## Materials and methods

Consider an infected person in an indoor environment who speaks, breathes, coughs, and sneezes, releasing droplets in the air. Suppose a person inhales a typical number of droplets (${N}_{d}$), each of which is expected to contain an average number of virions, ${n}_{v}$, then the risk of depositing at least one virion is then given by $R=1-exp\left({-N}_{d}{n}_{v}\right)$ according to the single Poisson fluctuation or single-hit model^7,33–38^, which implies that there are fluctuations in the number of virions in the droplet but not in the number of droplets inhaled. Also, the single-hit model assumes that every inhaled pathogen acts independently and has an individual probability of causing an infection^35,38^. The number of droplets inhaled would, however, fluctuate around the mean value at low droplet concentrations. As a result, the risk formula is modified to factor for both fluctuations (single-hit double Poisson model), and the modified risk for inhalation of at least one droplet carrying a virion can be expressed as,

2 $${R}^{^{\prime}}=1-exp\left({-N}_{d}\left[1-exp\left(-{n}_{v}\right)\right]\right).$$

The main difference is that in the modified formula (Eq. 2), the values ${N}_{d}$ and ${n}_{v}$ appear separately, rather than as a product, which is commonly employed in the literature^7,8,10–12,16^. A fundamental difference between the single Poisson model (SPM) and double Poisson model (DPM) is illustrated in a simple way in the Supplementary Information. The Supplementary Fig. S1 shows how the DPM deviates as the viral content in the droplet increases for various specified risks predicted by SPM for 1-hit risk case. For example, in the case of a single-hit SPM risk of 0.9, the DPM risk agrees with it only for very low viral content in the droplet (to preserve the total viral intake, the number of droplets inhaled must be large). However, as the viral content in drop increases, the DPM predicts lower than SPM risks. The differences are nontrivial, even around a mean viral content of one virion per drop. Also, the effect of the double-hit hypothesis in the estimation of infection risk is demonstrated in the Supplementary Fig. S2. In the present study, the above formula has been combined with averaging over the polydisperse size distribution function. The present double Poisson model has the advantage of being applicable even in the case of extremely low risk scenarios, such as inhalation for a brief period or at low droplet number concentrations.

By applying the "Falling-to-Mixing-Plate-out" model^21^ to the modified risk formula (Eq. 2), the infection risk for inhalation of at least one droplet carrying a virion (single-hit hypothesis) by a susceptible in the room environment is given by,

3 $${R}^{\mathrm{^{\prime}}}=1-{P}_{0}=1-{e}^{-k{C}_{0}\left\{{\int }_{{d}_{p}^{min}}^{{d}_{p}^{max}}{f}_{r}\left({d}_{p}\right){F}_{L}\left({d}_{p}\right)\left(1-{e}^{-\mu }\right)d{d}_{p}\right\}},$$

where ${P}_{0}$ is the probability of zero-hit, $k={PF}_{1}{PF}_{2}\dot{{q}_{B}}{t}_{expos}$ is a constant, ${C}_{0}=\frac{{S}_{0}}{{V}_{room}}{\int }_{0}^{\infty }\frac{{f}_{e}\left({d}_{w}\right)}{{\lambda }_{eff}}{dd}_{w}$ is total number concentration of original droplets suspended in the room, ${f}_{r}\left({d}_{p}\right)$ is the size distribution of the residues that undergone evaporation in the room, ${F}_{L}\left({d}_{p}\right)$ is the lung deposition fraction, $\mu =\frac{\pi }{6}{{\gamma }^{3}d}_{p}^{3}{C}_{v}^{^{\prime}}$, and ${d}_{p}$ is the particle diameter, ${d}_{p}^{min}$ and ${d}_{p}^{max}$ are the minimum and maximum diameter of the evaporated droplet considered for the calculations. In Eq. (3), the double Poisson fluctuation theory is introduced to account for statistics on droplet fluctuation as well as the probability of virus incorporation in droplets (virusolization). Equation (3) calculates the infection risk, which is a measure of the disease's reproduction number and transmissibility for a given viral load apart from the person's disease state and variant type (old wild, Delta or Omicron). This modified risk model is implemented in Mathematica^39^ and the findings are presented in the Results and Discussion sections for various exposure scenarios.

${PF}_{1}$ and ${PF}_{2}$ are the protection factors due to the masks/face shields worn by the infected and healthy person respectively, provide additional measures (deposition of emitted droplets) in reducing the spread of infection from both the emitter and receiver sides. $\dot{{q}_{B}}$ is the breathing rate of the healthy person, ${t}_{expos}$ is the exposure time, and ${V}_{room}$ is the room volume. ${S}_{0}$ is the total droplet emission rate, ${f}_{e}\left({d}_{w}\right)$ is the exhaled droplet number-size distribution, and ${d}_{w}$ is the wet droplet diameter. $\gamma$ is the ratio of wet droplet diameter $\left({d}_{w}\right)$ to evaporated droplet (residue) diameter $\left({d}_{p}\right)$. The droplet residue size is determined by the droplet composition and their mass. It is noted to be here that the all dynamics of settling is controlled by the size of the residue but the airborne virion concentration is prescribed by the wet droplet size.

**Figure 6:**
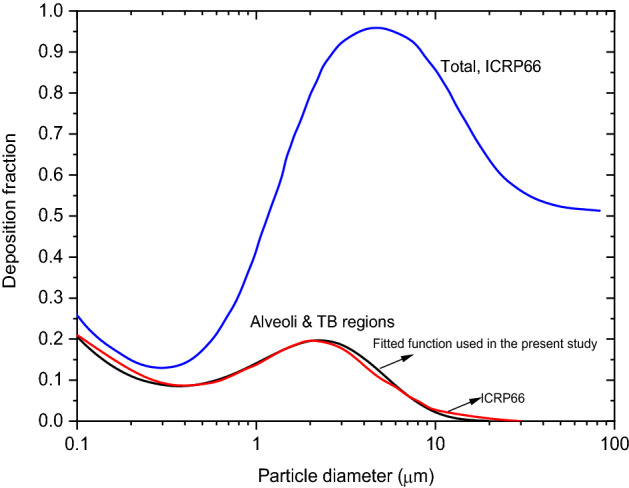
Particle deposition fraction as a function of particle diameter—comparison with ICRP model values for total, and alveoli and TB regions.

${C}_{v}^{^{\prime}}={C}_{v}{C}_{i}\frac{{\lambda }_{eff}}{{\lambda }_{d}+{\lambda }_{eff}}$ is the virion concentration corrected for its decay (inactivation), ${\lambda }_{d}$ is the inactivation rate of pathogens^40^, ${C}_{i}$ is the infectivity parameter (viable fraction) which accounts for the fact that not all pathogens will initiate/trigger the disease but only a fraction of pathogens will lead to infection, and ${C}_{v}$ is the viral load in the biological fluid. The natural decay of virion causes differential dilution of viral load in droplets, and plays an important role in limiting the residence time in certain cases and its inclusion in the model is essential. ${\lambda }_{eff}$ is the effective removal rate due to ventilation^41^, gravitational settling, bulk diffusion^42^ and wall deposition. The model takes into account the effect of ventilation caused by one or more of the following elements: fan, air cleaner, and natural air exchange. The deposition of residues in the respiratory system of a susceptible person is obtained by multiplying the residue number concentration distribution from the indoor model by the lung deposition probability function in the bronchial and pulmonary region given by,

4 $${F}_{L}\left({d}_{p}\right)=\left(0.0315{d}_{p}^{-0.9}+0.48{d}_{p}^{1.85}\right){e}^{-1.29{d}_{p}^{0.76}},$$

where ${d}_{p}$ is the particle diameter in μm. Equation (4) is constructed by fitting the deposition fraction curve from ICRP 66^15^ between 0.1 and 30 μm (Fig. 6).

To demonstrate the applicability of this model, a standard inhalation infection risk problem^7^ is used, in which four discrete droplet diameters (4.2 μm (1200 droplets), 9.0 μm (100 droplets), 14.6 μm (6.2 droplets) and 18.8 μm (1.7 droplets)) are released during coughing event (10 h^−1^) in room volume of 50 m^3^ with an air-exchange rate of 0.5 h^−1^. In this scenario, a viral load of (5 × 10^6^–5 × 10^10^) #/mL in the biological fluid is considered, with 0.1 h^−1^ inactivation rate. The risk estimates from the current model (Eq. 2) are compared to those of Nicas et al.^7^ using these input values (Table 3).

**Table 3:** Comparison of risk with Nicas et al.^7^.

Initial droplet diameter, µm	Single-hit risk					
	*C*_*v*_ = 5 × 10^6^ mL^−1^		*C*_*v*_ = 5 × 10^8^ mL^−1^		*C*_*v*_ = 5 × 10^10^ mL^−1^	
	Present model	Nicas et al.^7^	Present model	Nicas et al.^7^	Present model	Nicas et al.^7^
4.2	4.89E−04	1.93E−03	4.73E−02	1.76E−01	8.97E−01	1.00E+00
9.0	5.21E−03	5.89E−03	3.81E−01	4.46E−01	9.47E−01	1.00E+00
14.6	1.03E−03	9.07E−05	6.99E−02	9.03E−03	1.27E−01	5.96E−01
18.8	4.00E−04	4.62E−06	1.96E−02	4.62E−04	2.42E−02	4.52E−02

When compared to Nicas et al.^7^, the projected risk from the current model for the 4.2 μm droplet is four times lower for ${C}_{v}\le$ 5 × 10^8^ mL^−1^ (Table 3), owing to the difference in final droplet size, which determines its lifetime and lung deposition characteristics. In the case of larger size droplets (14.6 μm and 18.8 μm), the risk estimates from these two models differ by a ratio of ~ (8–90). According to the current simulation results, the final droplet diameter is decreased to ~ 1/5th of the original droplet (Fig. 1) as opposed to 50% reduction in Nicas et al.^7^ due to evaporation. The difference in the equilibrium droplet size is mainly due to the solid content in the saliva/droplet apart from other ambient conditions. A recent study by Lieber et al.^43^ shows that a mass concentration of salts and proteins of 0.8% in the saliva droplets will result in a ratio between equilibrium and an initial diameter of 20%. In the present work, a solid content of 8 g/L is assumed as against 88 g/L in Nicas et al.^7^, and the evaporation of droplet is modelled precisely and coupled with the other processes seamlessly.

The difference in the final droplet size leads to different sedimentation velocity that modifies the residence time of droplets in the indoor environment, and lung deposition fraction to estimate the inhalation risk. Also, the fluctuations in the low droplet number concentration (for 14.6 μm and 18.8 μm sizes) contribute to the variation in the risk estimation. In the case of higher viral load, the risk estimates from these two models are closer since $1-exp\left(-\mu \right)$ tends to 1 due to high viral load, and other effects compensate each other. Also, Nicas et al.^7^ assume that the droplets released are instantaneously mixed in the room environment and hence the concentration is uniform, whereas the present studies include the effect of ventilation induced turbulence to simulate the dynamics of the droplets in the room. Although final risk estimates from these two models are nearly the same in some cases, a large difference is observed in handling individual processes. Hence, it is recommended to couple the physical processes as much as possible and run the dynamic model to arrive at realistic estimates.

## Supplementary Information

Supplementary Information.

## Data Availability

All data generated or analysed during this study are included in this published article and its Supplementary Information files.

## List of symbols

${C}_{v}$: Viral load in the biological fluid, RNA copies/mL
$${C}_{i}$$: Infectivity parameter
$\tau$: Droplet residence time, s
$$RH$$: Relative humidity (50–90%)
$AER$: Air exchange rate, h^−^^1^
${S}_{0}$: Total droplet emission rate, #/s
${R}_{e}$: Event reproduction number
$R$: Infection risk for inhalation of at least one virion (single-hit hypothesis) corresponding to single Poisson model
${R}^{^{\prime}}$: Infection risk for inhalation of at least one droplet carrying a virion (single-hit double Poisson model)
${P}_{0}$: Probability of zero-hit
${P}_{0,B}$: Probability of zero-hit for breathing
${P}_{0,Sp}$: Probability of zero-hit for speaking
${P}_{0,C}$: Probability of zero-hit for coughing
${P}_{0,Sn}$: Probability of zero-hit for sneezing
${d}_{w}$: Wet droplet diameter, µm
${d}_{p}$: Evaporated droplet (residue) diameter, µm
${d}_{p}^{min}$: Minimum evaporated droplet (residue) diameter, µm
${d}_{p}^{max}$: Maximum evaporated droplet (residue) diameter, µm
$\gamma$: Ratio of wet droplet diameter $\left({d}_{w}\right)$ to evaporated droplet (residue) diameter $\left({d}_{p}\right)$
${C}_{0}$: Total number concentration of original droplets suspended in the room, #/m^3^
${f}_{r}\left({d}_{p}\right)$: Size distribution of the residues that undergone evaporation in the room
${F}_{L}\left({d}_{p}\right)$: Lung deposition fraction in the bronchial and pulmonary region
${C}_{v}^{^{\prime}}$: Virion concentration corrected for its decay (inactivation), #/m^3^
${\lambda }_{d}$: Inactivation rate of pathogens (0.63 h^−^^1^)
${\lambda }_{eff}$: Effective virusol removal rate due to ventilation, gravitational settling, bulk diffusion and wall deposition, h^−^^1^
${N}_{d}$: Typical number of droplets inhaled by a person
${n}_{v}$: Average number of virions in a droplet
${PF}_{1}$: Protection factor due to the masks/face shields worn by the infected person (0.1)
${PF}_{2}$: Protection factor due to the masks/face shields worn by the healthy person (0.1)
$\dot{{q}_{B}}$: Breathing rate of the healthy person (10 lpm)
$${t}_{expos}$$: Exposure time
$${V}_{room}$$: Room volume (100 m^3^)
${f}_{e}\left({d}_{w}\right)$: Exhaled droplet number-size distribution
$${T}_{amb}$$: Ambient temperature (25 °C)
$${T}_{d}$$: Droplet temperature (35 °C)
$CMD$: Count median diameter
$GSD$: Geometric standard deviation
$${Z}_{1}$$: Room height (4 m)
$$H$$: Release height (1.5 m)
