# Supplementary material for: A comprehensive modelling approach to estimate the transmissibility of coronavirus and its variants from infected subjects in indoor environments
Source: Sci Rep. 2022 Aug 19;12:14164. doi: 10.1038/s41598-022-17693-z (PMC9389491; doi:10.1038/s41598-022-17693-z)
Supplement: Supplementary file 1 — Supplementary Information. [file 41598_2022_17693_MOESM1_ESM.docx]

**Supplementary Information (SI)**

A comprehensive modelling approach to estimate the transmissibility of coronavirus and its variants from infected subjects in indoor environments

S Anand^1,3^, Jayant Krishan^1,3^, B Sreekanth^2,3^, Y S Mayya^4,*^

^1^Health Physics Division, ^2^Radiation Safety and Systems Division, Bhabha Atomic Research Centre, Mumbai, India. PIN – 400 085

^3^Homi Bhabha National Institute, Mumbai, India. PIN – 400 094

^4^Department of Chemical Engineering, Indian Institute of Technology Bombay, Mumbai, India. PIN – 400 076

*Corresponding author:

Prof. Y S Mayya,

Phone: +91-22-25767228

E-mail: ysmayya@iitb.ac.in

**Keywords**: aerosol, COVID-19, coronavirus, risk

**Table S1:** Comparison of infection risk models

|  | **Nicas et al.**^7^ | **Buonanno et al.**^8^ | **Buonanno et al.**^16^ | **Dhawan et al.**^12^ | **Peng et al.**^10^ | **Mizukoshi et al.**^9^ | **Azuma et al.**^17^ | **Sussman et al.**^13^ | **Netz et al.**^11^ | **Present study** |
| --- | --- | --- | --- | --- | --- | --- | --- | --- | --- | --- |
| Emission | Coughing, sneezing | Breathing at different conditions | Speaking, breathing, counting | Sneezing, coughing, speaking | Speaking, breathing, singing, exercise | Coughing, speaking | Coughing, speaking | Breathing, Speaking, Coughing | Speaking | Breathing, Speaking, Coughing, Sneezing |
| Size distribution | Discrete | Discrete | Total volume | Continuous | Total volume | Discrete | Discrete | Total volume | Continuous | Continuous |
| Virusol | Same viral load as the sputum | | | | | | | | | Poisson distribution |
| Evaporation of droplets | Parameterization, 50% reduction assumed | Not considered | Dehydrated volume is considered | Calculated for each size | Not considered | Not considered | Not considered | Started with evaporated droplet nuclei | Calculated for each size | Calculated for each size |
| Ventilation effect | Uniformly mixed, first-order rate | Uniformly mixed, first-order rate | Uniformly mixed, first-order rate | Diffusion, gravitational settling, and ambient air flow | Uniformly mixed, first-order rate | Uniformly mixed, first-order rate | Uniformly mixed | Uniformly mixed | Settling under gravity with evaporation | Falling-to-Mixing-Plate-out model |
| Gravitational settling | Stirred settling formula | Constant, 0.24 h^-1^ | Constant, 0.24 h^-1^ |  | Neglected | First-order rate | - | **-** |  |  |
| Airborne inactivation | Yes, first-order rate | Constant, 0.63 h^-1^ | Constant, 0.63 h^-1^ | Considered | Neglected | Considered | Yes + sterilization | Considered | Neglected | Considered |
| Air-cleaning factor | Yes, first-order rate | No | No | No | Yes, first-order rate | No | No | No | - | - |
| Respiratory deposition | Yes | No | No | Yes, ICRP model | No | No | No | No | No | Yes, ICRP model |
| Risk model | Single-hit & multiple-hit | Single-hit | Single-hit | Single-hit | Single-hit | Single-hit | Single-hit | Single-hit, | No | Single-hit & multiple-hit |
| Infectivity factor | No | Yes | Yes, PFU | Yes | Quanta emission | Yes, PFU | Quanta emission | Infective quanta | No | Yes |
| Variation of input parameters | Constant | Constant | Probability density function | Yes, for some parameters | Yes | Yes | Yes | Yes | Yes | Yes |
| Protection factor | No | No | No | Mask & distance | Mask | Mask | No | Discussed qualitatively | No | Yes |
| Re-suspension | No | No | No | No | No | No | Introduced | No | No | No |

**Comparison of single Poisson and double Poisson models with single- and double-hit approaches for the estimation of infection risk**

A fundamental difference between the single Poisson Model (SPM) and double Poisson model (DPM) can be illustrated in a simple way. Let us imagine that a patient emits $S_{0}$ (corrected for a facemask protection factor of the patient, *PF_1_*) monodisperse droplets of diameter ($d_{w}$) per unit time, each carrying an expected number of virions/mL, $C_{v}$. These emitted droplets get distributed in the room and assume a steady number concentration level ${(C}_{0})$ at a post-evaporative monodisperse residue diameter $d_{p}=\gamma d_{w}$, where $\gamma$ is a shrinkage factor. Each of these airborne residue particles is expected to carry $\mu_{v}=\frac{\pi d_{w}^{3}}{6}C_{v}$number of virions. Let a susceptible person inhale the residue particles at a breathing rate of $q_{B}$ for a time $T_{B}$ and let it lead eventually to deposition of “expected number” of particles ($N_{d}$) in the respiratory tract (RT). To be specific, $N_{d}=PF_{2}. f_{L}.C_{0}.q_{B}.T_{B}$, where $PF_{2} and f_{L}$ account for subject’s mask protection factor and lung deposition fraction. Finally, let $\mu_{D}$ denote the expected number of virions deposited in RT, and $\mu_{D}$ will be the product of the mean number of virions per particle deposited and the number of particles deposited,

i.e., $\mu_{D}=\mu_{v}.N_{d}$. (1)

Now, in the Riley single Poisson model, Poisson fluctuations are introduced only around the virion loading in the droplet (i.e., around $\mu_{v})$ and not in the fluctuations in the droplet deposition $N_{d}$. Hence, the risk becomes a unique function of the quantity $\mu_{D}$, which represents the “expected (mean) number of virions deposited in the RT”. For a single-hit model (i.e., just one virion deposited causes the risk of infection)

$R_{1SPM}=1-e^{-\mu_{v}.N_{d}}=1-e^{{-\mu}_{D}}$. (2)

Thus, in the single Poisson model, the risk is a unique function only of the number of active virions deposited ($\mu_{D}=\mu_{v}.N_{d})$ and not independently of the number of droplets deposited $(N_{d})$ or the active virions per droplet ($\mu_{v})$.

Since droplet intake by inhalation is also a discrete event, one would expect fluctuations in $N_{d}$ in addition to that around $\mu_{v}$. This would be more pronounced in situations where the droplets are sparse in number, but are larger in size and/or the viral loading is high. A rigorous derivation of this joint fluctuation through generating function method for 1-hit DPM yields a modified formula:

$R_{1DPM}=1-e^{{-N}_{D}(1-e^{-\mu_{v}})}$ (3)

instead of Eq.(2). Thus, in the double Poisson model, the risk is not a unique function of $\mu_{D}$ but involves both $N_{D}$ and $\mu_{v}$ explicitly. Only when $\mu_{v}\ll1$, Eq.(3) goes over to Eq.(2). Hence in high viral loading cases, one can expect a significant difference.

The generating function theory allows one to expand on the double Poisson model to a general risk of n-hit model. For example, if a minimum of two active virions are required to cause the disease, then the 2-hit risks as per SPM and DPM are:

$R_{2SPM}=1-\left( 1+\mu_{v}.N_{d} \right)e^{{-\mu}_{v}.N_{d}}$ (4)

$R_{2DPM}=1-\left( 1+\mu_{v}.N_{d}e^{{-\mu}_{v}} \right)e^{{-N}_{D}(1-e^{-\mu_{v}})}$ (5)

**Results:**

The comparison of these models is shown in Figs. S1 and S2. The first Fig. S1 shows how the DPM deviates as the viral content in the droplet increases for various specified Risks predicted by SPM for 1-hit risk case. For example, the topmost plot corresponds to 1-hit SPM Risk of 0.9 and the DPM risk agrees with it only for very low viral content in the droplet (to preserve the total viral intake μ_D_, the number of droplets inhaled must be large). However, as the viral content in the drop increases, the DPM predicts lower than SPM risks. The differences are nontrivial, even around a mean viral content of 1 virion per drop. This can be easily met by a 20 μm VMD drop carrying 10^11^ virion/mL.

**Figure S1: Comparison of single and double Poisson models for 1-hit risk model**

The second Fig. S2 is a comparison of DPM with SPM for 2-hit risk; these calculations correspond to 1-hit risk of 0.25. Here, the difference is even more visible. The $R_{2DPM}$ has non-monotonic behaviour with respect to μ_v_.

**Figure S2: Comparison of single and double Poisson models for 2-hit risk model**
